# Urinary high molecular weight matrix metalloproteinases as non-invasive biomarker for detection of bladder cancer

**DOI:** 10.1186/1471-2490-13-25

**Published:** 2013-05-14

**Authors:** Mohammed A Mohammed, Manar F Seleim, Mohga S Abdalla, Hayat M Sharada, Abdel Hady A Abdel Wahab

**Affiliations:** 1 Department of Cancer Biology, National Cancer Institute, Cairo University, Cairo, Egypt; 2 Department of Chemistry, Faculty of Science, Helwan University, Helwan, Egypt

**Keywords:** Bladder cancer, High molecular weight matrix metalloproteinases, Early detection, Biomarkers

## Abstract

**Background:**

Matrix Metalloproteinases (MMPs) are key molecules for tumor growth, invasion and metastasis. Over-expression of different MMPs in tumor tissues can disturb the homeostasis and increase the level of various body fluids. Many MMPs including high molecular weights (HMWs) were detected in the urine of prostate and bladder cancer patients. Our aim here is to assess the usefulness of HMW MMPs as non invasive biomarkers in bilharzial bladder cancer in Egyptian patients.

**Methods:**

The activity of different MMPs including HMW species was determined using zymographic analysis technique in the urine samples procured from sixty six bladder cancer patients (bilharzial and non-bilharzial) as well as hundred healthy control subjects. Also, the correlation between these HMW MMPs activities and different clinico-pathological parameters was investigated.

**Results:**

High frequency of urine MMPs (uMMPs) activity was determined in 63.6% of examined tumor cases, however, none of the control cases showed any uMMPs activity. MMP-9 had the highest activity (62%) followed by MMP9/NGAL (60%), MMP-2 (54.5%), MMP-9 dimer (53%), ADAMTS (25.6%), and the lowest one was MMP-9/TIMP-1 (12%) only. There was no correlation between uMMPs and any of clinico-pathological parameters including age, gender, tumor size and type, bilharziasis, grade, lymph node involvement, and invasion to the prostate. A significant correlation was established only between MMP-9/TIMP-1 activities with the tumor size.

**Conclusions:**

This study revealed that the detection of urinary MMPs including HMWs activity might be sensitive biomarkers for prediction of bladder cancer. It is also demonstrate that the detection of these urinary HMW gelatinases could not differentiate between bilharzial and non bilharzial bladder cancer subtypes.

## Background

Bladder cancer is the most common cancer in Egypt during the past 50 year [1,2]. The most common histopathological type of bladder cancer in Egypt is the squamous cell carcinoma (SCC), constituting from 59% to 81% between 1960–1981 [3]. Chronic bladder infection with *schistosoma haematobium* is the most important risk factor for bladder cancer [4]. Oncologists and pathologists have suggested that the ratio between SCC: TCC (transitional cell carcinoma) types of bladder cancer was changed over the past 10–15 years. This is because the change in the etiology of bladder cancer [5]. Definitive diagnosis of bladder cancer requires invasive cystoscopic examination. Despite urinary cytology is widely used for screening for bladder cancer, it is sometimes difficult to judge cytological specimens [6]. Therefore, non invasive and sensitive method is required for screening, diagnosis or even for monitoring of the disease.

The extracellular matrix (ECM) has a central role in organizing tissue architecture in animals and humans, providing the structural scaffold for cells. However, ECM is not merely a passive framework, but rather a dynamic system that interacts with cells and influences their functions such as cell proliferation, growth, differentiation, migration, adhesion and survival. Conversely, cells are also able to influence and reorganize their surrounding ECM. ECM-cell interaction is essential to maintaining the physiological microenvironment and homeostasis. Any disturbance in this complex regulation have been implicated in several pathological processes including unregulated tumor growth, invasion and metastasis [7]. Overexpression of MMPs in tumor tissues and stroma can increase level of MMPs activities in various body fluids. Multiple lines of evidence show that MMPs can be detected in urine samples procured from patients suffering prostate and bladder cancers and MMPs were independent predictor of disease status [8,9]. Several MMP species were detected in urine from cancer patients including MMP-2, MMP-9, MMP-9/NGAL. Several studies identified three high molecular weight gelatinases in urine of bladder cancer patients [10,11]. These MMPs were MMP-9 and its inhibitor TIMP-1 (140KDa), MMP-9 dimer (220KDa), and disintegrin and metalloproteinase with thrombospondin motifs-7 (ADAMTs-7) [12].

The objective of the current study is to identify the different gelatinases activity in the urine samples collected from bilharzial bladder cancer patients (in particular HMW gelatinases) and to correlate their activities with different clinico-pathological parameters of the disease.

## Methods

### Clinical features of bladder cancer patients

This study was conducted in a compliance with the guidelines of good ethical principles rooted in the Declaration of Helsinki. All patients consented to participate in the study. The study was approved by the Institutional Review Board of the Egyptian National Cancer Institute NCI-Egypt This study encompassed 66 bladder cancer patients presented to NCI-Egypt from April 2008 to May 2011. Another 100 healthy control groups without any history of cancer, inflammation, or immunodeficiency diseases were also included with average age of 51 ± 6 years (75 males and 25 females). The average age of bladder cancer group was 54 ± 8 years (48 males and 18 females). Transitions cell carcinoma type was about 68% of all patients, SCC was 32%. Grade II was the common histological grade of the tumor (56%) whereas GI and GIII were about 9.1% and 30% respectively. Tumors associated with bilharziasis were 33% and the free of bilharziasis were 67%. The percentage of positive lymph nodes was 6%. About 44% of the tumors had muscle infiltrated and about 15% had metastatic to prostate (Table 1).

**Table 1:** Clinical features of bladder cancer patients in the present study

**Clinical parameters**		**Bilharzial bladder cancer subgroup**	**Non- bilharzial bladder cancer subgroup**	**Total**
		**n = 22(%)**	**n = 44 (%)**	
**Gender**	**Male**	18 (27.3)	30 (45.5)	48 (72.7)
	**Female**	4 (6.1)	14 (21.1)	18 (27.3)
**Age (yrs)**	**≤ 60**	7 (10.6)	20 (30.3)	27 (40.9)
	**>60**	15 (22.7)	24 (36.4)	39 (59.1)
**Tumor size**	**≤ 5 cm**	4 (6.1)	24 (36.4)	28 (42.4)
	**>5 cm**	18 (27.3)	20 (30.2)	38 (57.6)
**Tumor type**	**TCC**	13 (19.7)	32 (48.5)	45 (68)
	**SCC**	9 (13.6)	12 (18.2)	21 (32)
**Tumor grade**	**1/2**	16 (24.5)	27 (40.4)	43 (65)
	**3/4**	5 (7.7)	15 (22.9)	20 (30)
	**Missing**	1 (1.5)	2 (3.0)	3 (5)
**Lymph-node**	**present**	2 (3)	2 (3)	4 (6)
	**Absent**	20 (30.4)	42 (63.6)	62 (94)
**Prostatic invasion**				
	**Present**	5 (7.6)	5(7.6)	10 (15.2)
	**Absent**	17 (25.8)	39 (59)	56 (84.8)

### Sample collection and processing

Fresh voided urine samples were obtained before surgical or other therapeutic intervention. Samples were collected in sterile containers and immediately frozen at -20°C. Urine was tested for presence of blood or leucocytes using urine analysis strips and the samples containing blood or leucocytes were excluded from the study. Protein concentration was measured in all urine supernatant by the Bradford method according to the manufacturer’s protocol (Bio-Rad, CA).

### Zymographic analysis

Gelatinases in the urine were detected using gelatin zymography as previously described [10]. Briefly, 40 μL urine samples from controls or cancer patients were mixed with non- reducing sample buffer (4% SDS, 0.15 M Tris, pH 6.8, 20% v/v glycerol, and 0.5% w/v bromphenol blue) and separated on a 10% polyacrylamide gel containing 0.1% gelatin (Bio-Rad, Hercules, CA). After electrophoresis, gels were washed twice with 2.5% Triton X-100 (15 min/each wash). Substrate digestion was carried out by incubating the gel in 50 mM Tris- HCl, pH 7.6, containing 5 mM CaCl_2_, 1 μM ZnCl_2_, 1% Triton X-100, and 0.02% NaN_3_ at 37°C for 24 h. The gel was stained with 0.1% Coomassie Brilliant Blue R250 (Bio-Rad). Molecular weight protein marker appeared as easily visible staining bands against the lighter blue color of 108 the stained gel background. Gelatinase activity was detected as zones of clearance on a background of uniform blue staining and imaged using densitometer (BIOMETRA).

### Statistical analysis

The patients were described using descriptive statistics functions. Nominal and categorical variables were summarized as percentages while numerical data as means and standard deviation. Sub-groups were compared using Chi Squared test when dealing with nominal or categorical variable and independent *t*-test or ANOVA when comparing numerical values. Groups were compared using the log-rank test. A Probability less than 0.05 (p < 0.05, two tailed) was considered statistically significant. The software SPSS (win version 13.0, SPSS Corporation, Chicago, Il, USA) was used in the analysis.

## Results

### Enzymatic activity of different MMPs in urine of bladder cancer

**Figure 1:**
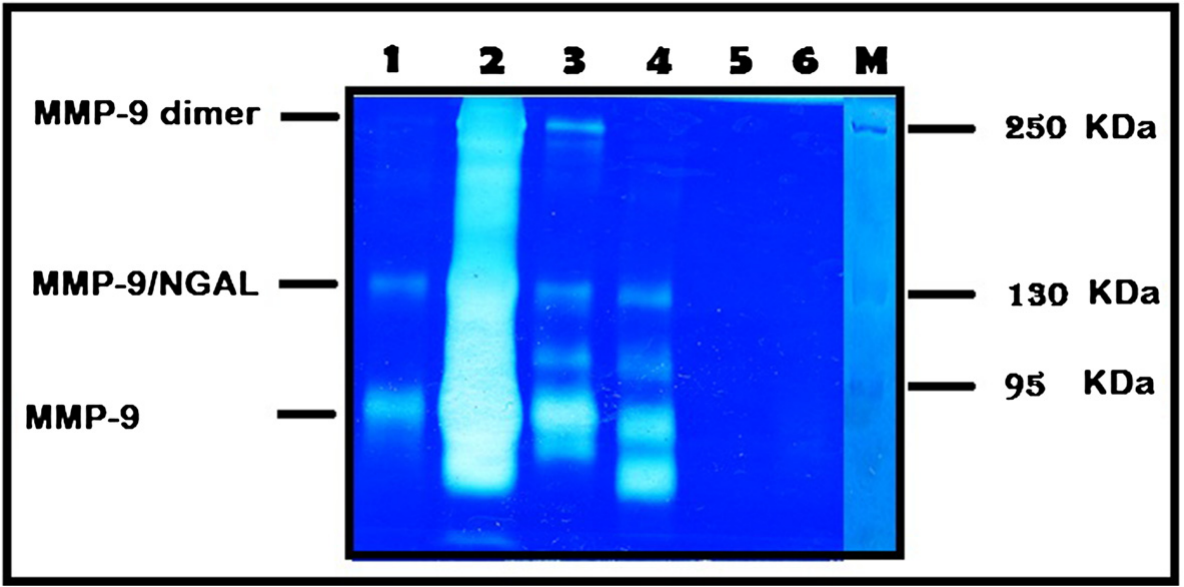
**Urine samples procured from bladder cancer patients were analyzed by substrate gel electrophoresis (zymography).** Bands of enzyme activity were detected as zones of clearance on a background of uniform blue staining. Protein marker used to detect the molecular weight. Arrows indicate HMW MMP9 dimer, MMP9-NGAL complex, MMP9. Urine samples from bladder cancer patients in lane (1–4) show activity for MMPs, while no activity in urine sample of control in lanes (5&6).

Matrix Metalloproteinases (MMPs) including high molecular weights were analyzed in urine of bladder cancer patients as well as 100 healthy control subjects using zymographic analysis technique. High frequency of tumor cases of different uMMPs (63.6%; 42 out of 66) showed activity whereas none of the healthy control cases showed any of the MMPs studied. A representative image of association was observed between each of MMPs detected in the urine of bladder cancer patients as compared with the controls (Figure 1). The highest percent of activity detected was for MMP9 (62%) among all investigated markers followed by MMP9/NGAL (60%), MMP2 (55%), MMP9/dimer (53%), the lowest percent of activity was observed for ADAMTS (25.6%) and MMP9/TIMP-1 (12.1%).

### Correlation between MMP-9 dimer with different uMMPs

In the current study, it was noticed that the highest percentage of activity of HMW MMPs detected was in MMP9 dimer (53%). This prompted us to find its correlation with other different uMMPs detected using multivariant analysis. Data analysis showed a highly significant correlation with the activity of MMP2, MMP9, and MMP9/NGAL. On the other hand, no correlation found between MMP9 and MMP9/TIMP-1 or ADAMTS activities as shown in Table 2.

**Table 2:** The correlation between activities of uMMP-9 dimer with different uMMPs examined in bladder cancer patients

**uMMPs**	**MMP-9 dimer (%)**		**Total**	**Measurement of agreement**	**p-value**
	**Negative**	**Positive**			
	**(31)**	**(53)**		**(kappa test)**	
MMP-2					
Negative	26 (83.9)	4 (11.4)	30		
Positive	5 (16.1)	31 (88.6)	36	0.726	<0.001
MMP-9					
Negative	24 (77.4)	1 (2.9)	25		
Positive	7 (22.6)	34 (97.1)	41	0.754	<0.001
MMP-9/NGAL					
Negative	25 (80.6)	1 (2.9)	26		
Positive	6 (19.4)	34 (97.1)	40	0.785	<0.001
MMP-9/TIMP					
Negative	29(93.5)	29 (82.9)	58		
Positive	2 (6.5)	6 (17.1)	8	0.102	>0.05
ADAMTS-7					
Negative	26 (83.9)	23 (65.7)	49		>0.05
Positive	5 (16.1)	12 (34.3)	17	0.176	

### Correlation between different uMMPs with clinic-pathological parameters

To assess the correlation between different uMMPs detected in the present study with several clinic-pathological parameters, Pearson Correlation was applied. The variables included were age, gender, tumor size, stage, grade, presence of bilharziasis, tumor type, lymph nodes, invasion to prostatic walls as summarized in Table 3. No significant correlation was found between the activity of different MMPs examined with the gender except in MMP9/TIMP-1 complex, which showed the trend of higher activity in male more than female patients (p = 0.063). Likewise, a positive correlation was also found between the activity of ADAMTS marker and the age at diagnosis. This correlation was higher in young age compared to elder group (p = 0.068). High expression of MMP2, MMP9, MMP9/NGAL, and MMP9/dimer was observed in high grade patients (3/4) as compared to low grades (1/2) but did not reach the significant values. Also, a non significant difference was reported between the activity of different MMPs with the tumor size except MMP9/TIMP-1, which showed a significant high expression in patients with large tumor size (>5 cm) as compared to low size group (p = 0.032).

**Table 3:** The correlation between the activities of different uMMPs with clinico-pathological parameters

**Clinic-pathological parameters**	**MMP-2**	**MMP-9**	**MMP-9/NGAL**	**MMP-9 dimer**	**MMP-9/TIMP**	**ADAMTS**
	**(%)**	**(%)**	**(%)**	**(%)**	**(%)**	**(%)**
Gender						
Male	56.3	65.6	62.5	54.2	16.7	25.0
Female	50.0	55.6	55.6	50.0	0.0	27.8
Age (year)						
≤ 60	47.8	52.2	56.5	47.8	34.8	21.7
> 60	58.1	67.4	62.8	53.5	20.8	7.0
Tumor size						
≤ 5 cm	52.8	58.3	58.3	52.8	16.7*	13.9
> 5 cm	58.3	70.8	66.7	50.0	41.7	12.5
Tumor type						
TCC	55.6	62.2	60.0	53.3	13.3	22.2
SCC	52.4	61.9	61.9	52.4	9.5	33.3
Grade						
1/2	48.8	58.1	53.3	48.8	16.3	27.9
3/4	65.0	70.0	75.0	65.0	5.0	20.0
Bilharziasis						
Present	59.1	72.7	68.2	45.5	18.2	36.4
Absent	52.3	56.8	56.8	56.8	9.1	20.5
Lymph nodes						
Present	25.0	75.0	75.0	25.0	50.0	50.0
Absent	52.4	61.9	59.5	54.8	14.5	28.6
Prostatic invasion						
Present	60.0	80.0	80.0	60.0	20.0	30.0
Absent	42.9	50.0	50.0	50.0	14.3	21.4

^*^ Statistically significant.

High expression of different uMMPs investigated with the presence of muscle invasion to prostate but this correlation was not significant enough. A non-significant increase in the activity of uMMPs was found towards bilharzial bladder cancer patients as compared to non bilharzial once. No association was observed with tumor type or lymph node involvement. We could not make a correlation between the activities of MMPs with tumor staging due to the insufficient biopsy for staging process.

### Sensitivity and specificity in MMPs markers

Sensitivity, specificity, accuracy, positive and negative values for each uMMPs as well as their combination was tested for detection of bladder cancer. The highest sensitivity was found in uMMP-9 and uMMP-9/NGAL (62.1% and 60.9%), respectively, followed by MMP-2 (54.5%), and MMP-9 dimer (53%) whereas the lowest recorded values was observed in ADAMTS (25.8%) and MMP-9/TIMP(12.1%). The specificity of each marker examined reached 100% where none of the normal healthy control cases showed any activity. A combination of each of the makers examined did not show any significant increase of the sensitivity as shown in Table 4.

**Table 4:** Sensitivity, specificity, accuracy, PPV and NPV for different uMMPs in bladder cancer patients

**Markers**	**Total (%)**	**MMP-2 (%)**	**MMP-9 (%)**	**MMP-9/NGAL (%)**	**MMP-9 dimers (%)**	**MMP-9/TIMP (%)**	**ADAMTS (%)**
Sensitivity	42/66	36/66	41/66	40/66	35/66	8/66	17/66
	(63.6)	(54.5)	(62.1)	(60.9)	(53)	(12.1)	(25.8)
Specificity	100/100	100/100	100/100	100/100	100/100	100/100	100/100
	(100)	(100)	(100)	(100)	(100)	(100)	(100)
Accuracy	142/166	136/166	141/166	140/166	135/166	108/166	125/166
	(85.5)	(81.9)	(84.9)	(84.9)	(81.3)	(65)	(70.4)
PPV	42/42	36/36	41/41	40/40	35/35	8/8	17/17
	(100)	(100)	(100)	(100)	(100)	(100)	(100)
NPV	100/124	100/130	100/125	100/126	100/131	100/158	100/149
	(80.6)	(76.9)	(80)	(79.4)	(76.3)	(63.3)	(67.1)

PPV: positive predictive value.

NPV: negative predictive value.

## Discussion

Detection of certain MMPs profile in urine of patients could provide invaluable information for the tumor biology features. The presence of different MMPs including HMWs (MMP9-dimer, MMP9-TIMP-1, ADAMTS-7) was reported in the urine of a variety of cancer patients including bladder [11,12]. Increase of MMP activities serve to facilitate both tumor cell migration and development of new tumor-related blood vessels [10]. The current study aimed to detect HMW-MMPs gelatinases in the urine of bladder cancer patients and not only to predict the disease onset but also to differentiate between bilharzial and non bilharzial bladder cancer. The activity of MMPs was detected in urine of 66 bladder cancer patients as well as 100 normal healthy controls using zymographic analysis technique. In our study, the activity of MMP-2 and MMP-9 recorded was 55% and 62%, respectively, and the higher activity of MMP-9 may be explained as the effect of transcriptional regulation by inflammatory cytokines such as TNFα and interlukins. MMP-2 is expressed by many cell types and most likely is regulated by the level of proenzyme activation [13]. Different independent studies consistently reported elevated MMP-2 activity in urine samples from bladder cancer as compared to normal healthy subjects, which confirm our results and raise the hope HMW MMPS can be used as a diagnostic biomarker for this malignancy [8,14,15]. On the other hand, the secretion of MMP-9 was enhanced by presence of inflammatory cells and so the presence of cystitis might lead to false positive results and decrease specificity [16]. In addition, low sensitivity of urinary MMP-9 was detected in bladder cancer, which was approximately 40% in two independent studies [14,16] including our study (62%), showing a limited diagnostic value of MMP-9 in the urine of such type of malignancy. The higher activity of both MMP-2 and MMP-9 were detected in high grades and muscle invasion to prostate gland and this may indicate their role in the progression of disease. Also, higher levels of gelatinases (2 & 9) in bilharzial bladder cancer patients compared to their counterpart control might have clinical relevance in the detection of this specific type of bladder cancer. The non – significant association between MMPs and other clinical parameters probably came from the limited number of cases examined. Our study warrants further investigations to clarify this finding.

When MMP-9 present in excess amount relative to its endogenous inhibitor TIMP, it can form dimmers. MMP-9 dimer has been identified in a variety of MMP-9 producing cells including neutrophils and normal breast epithelial cells [17,18] and is a component of normal plasma [19]. Enzymatic activity of the monomeric and dimeric forms of MMP-9 does not differ; however, the dimer can be activated by stromelysin with much lower efficiency (10 fold less) than monomer [19]. The existence of more stable, slow activating MMP-9 dimer might serve as a regulatory mechanism during extracellular matrix degradation [19]. In our study, MMP-9 was the most detectable gelatinases in the urine of bladder cancer patients which means a relative excess level of MMP-9 relative to TIMP-1 produced by the primary tumor and surrounding stroma. This in turn resulted in elevated levels of both monomeric and diametric forms of this protease in urine [12].

It was previously demonstrated that the ~125 kDa MMP activity in the urine of cancer patients is a complex of MMP-9 and NGAL [11], which may represent a new biomarker for the prediction of cancer disease [20]. Several studies reported an elevation of this enzyme activity in the urine of variety of cancer including bladder cancer [20,21]. The formation of NGAL-MMP-9 complex has the ability to protecting MMP-9 from autodegradation. MMP-9/NGAL complex was detected more frequently in the urine of metastatic cancer than other types of disease. It is believed that invasive capacity of MMP-9 is enhanced when it is bound to NGAL and that is associated with metastasis [11]. In the current study, MMP-9/NGAL enzyme activity was detected in about 60% of the urine specimens of the bladder cancer patients but not in any of the urine control healthy cases (p = 0.001). However, no marked difference was recorded in the ration of NGAL within bilharzial and non bilharzial bladder cancer patients or even between the TCC and SCC types. Likewise, MMP-2 and 9 had no significant difference in high grade and muscle invasion bladder cancer cases which confirm their role in the tumor progression.

Here, we report that the 190 kDa gelatinase (ADAMTS-7) was detected in the urine of a little number of bladder cancer patients (26%). To date, ADAMTS-7 has yet to be associated with human cancers except with those reported by Roy et al. [12]. ADAMTS-7 is a family of disintegrin zinc – dependent proteases that have at least one thrombospondin type I motif [12]. A non significant correlation was recorded between the enzymes activities with different clinico- pathological parameters.

MMP-9/TIMP-1 complex was reported to be serum marker for fibrosis in children with chronic hepatitis B [22]. However, very little is known about its correlation with cancer. In this study, we found that urine from bladder cancer patients indicated the presence of 140 kDa MMP- 9/TIMP-1 complex but it was unfrequented in urine of those patients.

Taken together, our study detected many species of gelatinases in the urine of bladder cancer patients with primary tumor (both bilharzial and non bilharzial) including MMP-2, MMP-9, MMP-9 dimer, MMP-9/NGAL, MMP-9/TIMP-1 and ADAMTS-7. Each MMP was detected at significantly higher rate in urine from cancer patients compared to control subjects except in MMP-9/TIMP-1 and ADAMTS-7. The activity of urinary MMPs is a promising diagnostic tool and can be used as biomarkers for predicting bladder cancer. The study also revealed that the detection of urinary HMW gelatinases was not able to differentiate between bilharzial and non bilharzial bladder cancer subtypes.

## Conclusions

This study revealed that the detection of urinary MMPs including HMWs activity might be sensitive biomarkers for prediction of bladder cancer. It is also demonstrate that the detection of these urinary HMW gelatinases could not differentiate between bilharzial and non bilharzial bladder cancer subtypes.

## Competing interests

All authors declared that they have no competing interests.

## Authors’ contribution

MAM: conceived the design of the study, participated in the practical part and interpretation of data, helped to draft, revise the manuscript, has involved in the final approval of the version for publication. MFS: carried out most of the practical part, performed the statistical analysis and interpretation of data. MSA: participated in design of the study, participated in the data analysis and interpretation of data, has involved in the final approval of the version for publication. HMS: participated in design of the study, participated in the data analysis and interpretation of data, has involved in the final approval of the version for publication. AAA: participated in the practical part and interpretation of data, writing and drafting the manuscript, has involved in the final approval of the version for publication. All authors read and approved the final manuscript.

## Pre-publication history

The pre-publication history for this paper can be accessed here:

http://www.biomedcentral.com/1471-2490/13/25/prepub
